# Evaluating tools to support a new practical classification of diabetes: excellent control may represent misdiagnosis and omission from disease registers is associated with worse control

**DOI:** 10.1111/j.1742-1241.2012.02979.x

**Published:** 2012-09

**Authors:** N Hassan Sadek, A-R Sadek, A Tahir, K Khunti, T Desombre, S de Lusignan

**Affiliations:** 1 Department of Health Care Management and Policy, Surrey UniversityGuildford, UK; 2 Department of Health Sciences, University of LeicesterLeicester, UK

## Abstract

**Aims:**

To conduct a service evaluation of usability and utility on-line clinical audit tools developed as part of a UK Classification of Diabetes project to improve the categorisation and ultimately management of diabetes.

**Method:**

We conducted the evaluation in eight volunteer computerised practices all achieving maximum pay-for-performance (P4P) indicators for diabetes; two allowed direct observation and videotaping of the process of running the on-line audit. We also reported the utility of the searches and the national levels of uptake.

**Results:**

Once launched 4235 unique visitors accessed the download pages in the first 3 months. We had feedback about problems from 10 practices, 7 were human error. Clinical audit naive staff ran the audits satisfactorily. However, they would prefer more explanation and more user-familiar tools built into their practice computerised medical record system. They wanted the people misdiagnosed and misclassified flagged and to be convinced miscoding mattered. People with T2DM misclassified as T1DM tended to be older (mean 62 vs. 47 years old). People misdiagnosed as having T2DM have apparently ‘excellent’ glycaemic control mean HbA1c 5.3% (34 mmol/mol) vs. 7.2% (55 mmol/mol) (p < 0.001). People with vague codes not included in the P4P register (miscoded) have worse glycaemic control [HbA1c 8.1% (65 mmol/mol) SEM = 0.42 vs.7.0% (53 mmol/mol) SEM = 0.11, p = 0.006].

**Conclusions:**

There was scope to improve diabetes management in practice achieving quality targets. Apparently ‘excellent’ glycaemic control may imply misdiagnosis, while miscoding is associated with worse control. On-line clinical audit toolkits provide a rapid method of dissemination and should be added to the armamentarium of quality improvement interventions.

**What's known**

- Disease registers are associated with improved quality of care
- There are problems with the coding, classification and diagnosis of diabetes
- Around 40% of the coding, classification and coding errors found on computer searches reflect suboptimal management

**What's new**

- On-line self-audit tools to correct coding, classification and diagnosis errors in diabetes are downloaded and usable.
- Where people with diabetes are not included in disease registers, through no coding or use of vague codes, their control is not as good.
- People with Type 2 diabetes misclassified as Type 1, tend to be older.
- People with excellent control of their diabetes may not actually have the disease at all.

## Introduction

Diabetes prevalence is rising, as is its associated morbidity and mortality, and this increase has led to the development of educational and treatment programmes to improve management (1–3). For over two decades many patients with diabetes have not received care as recommended in guidelines (4) despite a longstanding appreciation of the importance of adhering to national standards of care (5).

Primary care is now central in the management of chronic diseases, such as diabetes, in that the numerous programmes that are *in situ* are implemented correctly. In the UK there has been a system of pay-for-performance (P4P) to drive up quality in diabetes management since 2004, with an update in 2006 requiring separate disease registers for people with T1DM and T2DM. UK primary care has a registration based system, every citizen is registered with a single practice making it feasible for quality improvement interventions to act at the population level (5). In addition, nearly all practices are computerised with all P4P data collected automatically from routinely collected data (6). Despite this there are problems with the misdiagnosis, misclassification and miscoding of diagnostic codes associated with diabetes (7,8,17) with possible resultant reduction in quality of care. Hence, the Royal College of General Practitioners (RCGP) and NHS Diabetes have launched a Classification of Diabetes (CoD) programme, which includes self-audit tools to allow practices to identify likely cases of misdiagnosis, misclassification and miscoding of diabetes (7,9). These self-audits are freely available for practices to download (10) and the results from the pilot use of these audit tools shows they identify clinically important cases (Table 1) (11).

**Table 1:** Characterising people who are misclassified and misdiagnosed; and the difference in glycaemic control in people miscoded and not part of P4P disease registers

Summary of audit data	

Finding	Quantitative basis of finding
Older T2DM people are more likely to be misclassified as T1DM	Mean age 62 years vs, 47 years for people with true T1DM
Misclassified T1DM people have lower HbA1c than true T1DM	True T1DM 8.5 vs 7.7 misclassified T1DM (paired *t* test p = 0.029)
Correctly diagnosed T2DM people tend to have increases in weight and falls in their HbA1c	BMI increases from 28.4 to 29.2 (not significant), HbA1c falls from 5.7 to 5.3 (p < 0.001)
Miscoded people are managed suboptimally	Mean HbA1c significantly lower in patients on the disease register (HbA1c, SEM 0.11 vs. HbA1c 8.1 SEM = 0.42, p = 0.006)
Those people on a disease register have significant improvements in their HbA1c reduction	From HbA1c 7.6 (SEM = 0.14) to 7 (SEM = 0.12) *t* test p < 0.001)

The audit with the accompanying downloadable toolkit was carried out in eight volunteer practices, five in Surrey and three in southwest London. The practices had a combined list size of 72,000 and a mean of 9000 patients; median 10,043. The practices had all created a disease register of people with diabetes, as part of P4P performance quality targets. The disease registers contained a total of 2340 people with diabetes, representing an overall prevalence of 3.2% (range 2.9–3.9%).

The practices had all created a disease register of people with diabetes, as part of pay-for-performance (P4P) quality targets. These disease registers contained a total of 2340 people with diabetes, representing an overall prevalence of 3.2% (range 2.9–3.9%).

We carried out this service evaluation to report the strengths and weaknesses of this approach to quality assessment. We wanted to know if practices could download and run the online audit tools we have developed. We also wanted to know if clinicians found the process clinically relevant; including an exploration as to whether those who sit outside the P4P quality targets or are misclassified are receiving a lower standard of care.

## Method

### Introduction

We carried out a process evaluation to describe the quality of the intervention, and the experience of those exposed to it (12).

### Setting

Eight practices volunteered to take part in the audit, and two offered undertake it without training to allow the audit process to be observed (11). We wanted to observe the process of going to the web-site (http://www.clininf.eu/cod/), finding the online tools, downloading and running the searches, and the process of sorting the cases for clinical review – we had no interest in direct observation of the clinical records; and no clinical records or identifiable data were viewed or removed from the practices. All the practices participating were at or very close to the maximum pay-for-performance (P4P) indicator for diabetes chronic disease management (18).

### Qualitative appraisal

We carried out the qualitative elements of this evaluation using participant observation; (4) a widely used method (16). The practices that volunteered to take part in the audit felt that time was the principal barrier to participation and would welcome assistance; NS therefore agreed to be a participant observer. NS was both naive of the data collection method, and had had very little prior exposure to clinical audit (NS was a medical student on elective at the time of this audit).

We observed and ran the audit in two practices, and NS made notes on the search process and the reactions of practice colleagues, documenting these in a field book. The practice staffs were observed throughout implementation of the audit.

### Identifying problems

We wanted to highlight any difficult steps in the audit and identify strategies and workarounds developed to overcome them. We applied a standard error reporting taxonomy to classify the type of problems that took place (19). The audit tools came with a step-by-step manual of how to complete the audit, following a pattern we had successfully used to create searches for health service localities wanting to assess the numbers of cases likely to need access to new psychological therapy service (20). However, the psychological therapy service audit was designed to be used by health service managers assisted by clinical audit teams. The nearest we had previously produced for use in practice was a tool for converting serum creatinine readings into estimated glomerular filtration rate, needed to diagnose chronic kidney disease; (21) although much used this was considerably simpler (22).

### Overcoming barriers

We decided to offer multimedia approaches to overcome any problems identified. We offered to create: graphics, video, or audio files should that be needed to overcome any obstacles in the audit process. We decided that should this be necessary we would simulate the relevant steps in a way that no patient information would be displayed. We felt that any such anonymous help should be made available through publically available media such as YouTube.

### Feedback

There were three elements to the feedback: firstly the direct comments from the audit practices; secondly the number of downloads in the 2 months following the launch; and finally we reviewed the problems that arose running the searches. On completion of the pilot audit, we asked participating practices to comment on their experience informally or via the audit in a comment box on our website. In addition, download statistics on the number of downloaded toolkits were collected. Informal feedback both verbal and via email was also obtained. It was noted that the vast majority of the participants in the pilot audit were grossly satisfied with the provision of an audit toolkit with the primary hindrance linked to poor digital patient record keeping.

We also agreed to revisit data from a wider group of eight audit practices to help clarify any questions that arose as part of the audit. The eight audit practices comprised six other practices who had volunteered to participate in the audit in addition to the two in-depth evaluation practices (11).

### Data analysis

To analyse our findings we used the following statistical tests for our audit; Pearson χ^2^, an independent samples *t*-test and a paired sample *t*-test. All the analysis was performed on SPSS (PASW/IBM statistics) version 18 software.

### Ethics

This audit was carried out to improve the quality of classification of diabetes as recommended by WHO (7). The practices volunteered in accordance with the General Medical Council (8) guidance to participate in local audit. This service evaluation is congruent with the National Research Ethics Service (5) definitions. Identifiable data were held in individual practices only; and anonymised data were held at St George’s; only able of being re-identified in contributing practices.

## Results

### Qualitative findings

#### User experience

Clinical audit naïve staff ran the audits satisfactorily with a number of features within the toolkit contributing to its success. NS reported, as a non-experienced user of an electronic patient record (EPR) system that running the searches was straightforward. The accompanying detailed user guide provided with the audit toolkit made this possible through step-by-step screen shots and annotations. Once the relevant cases had been highlighted in the analysis spread sheet and patient information had been retrieved from the computerised medical records, data entry into the audit worksheet was manageable.

A number of challenges potentially limit the audit process:

1. Lack of an overview. Whilst the ‘logic’ explanation under each query helped with understanding what the individual searches were trying to achieve, the process itself was conducted step-by-step without any initial clear overview. This could create confusion in people with little background knowledge of the aim of the audit.
2. Poor electronic and paper record keeping of general practitioners also hindered the process. In one of the practices audited, there were no medical records for some patients pre-2005, making full audit implementation impossible.
3. Practice staff members wondered whether misclassification mattered. Specific questions arose from the practices in the planning of the audit: firstly what characterised a patient who was misclassified, and secondly the *so what* question: does it really matter if patients are miscoded and not included in the P4P register; summary data from the audit are shown in Table 1.
4. There general was surprise from the practitioners involved in the audit that ‘real’ problems were identified. They reported that as they were achieving maximum or near maximum P4P they did not expect there to be problems with the quality of their diabetes coding.

**Figure 1:**
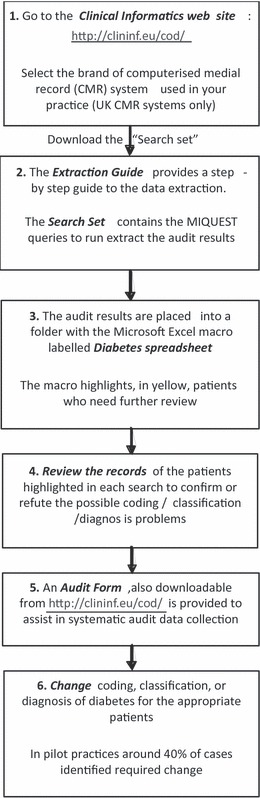
Overview of audit process

**Figure 2:**
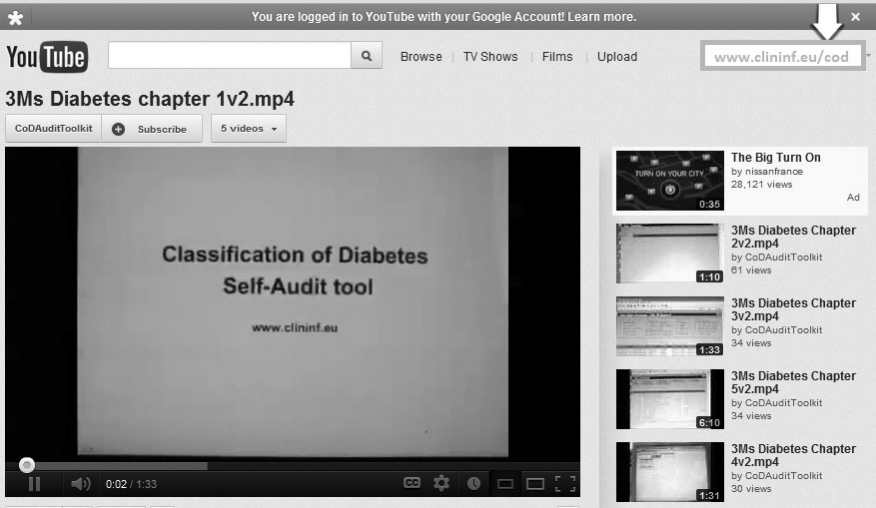
YouTube video illustrating the process of running the audit toolkit (http://www.youtube.com/user/CoDAuditToolkit#p/u)

We responded to the first challenge by creating an overview, which we made available on-line with the toolkit (Figure 1, and online at: http://www.clininf.eu/images/stories/cod/audit_toolkit_overview_flowchart.pdf). We also created an on-line video of the process at You tube;(23) dividing the audit process into five sections, each described in a separate video. However, this has been little used – one was downloaded five times, the others once (Figure 2).

We could not help with the second challenge (point 2); only searching out the written notes could help further here and practices felt they did not have time to do this. Finally, we addressed the latter two points by comparing the people who were misclassified, misdiagnosed and miscoded with those who were not to see if this provided further insight as to whether this ‘mattered;’ using data from all eight participant practices (Table 1).

We were able to characterise people misclassified as having T2DM when they really had T1DM as older, and likely to have achieved good glycaemic control. Patients who were misclassified as having T1DM when they had T2DM were more likely to be older (mean age 62 years vs. 47 years for people with ‘true’ T1DM). They tend to show a more substantial reduction in HbA1c than people with T1DM. [True T1DM 8.5–7.7 (n.s. p = 0.18) vs. T2DM misclassified as T1DM 9.1–7.6 (p = 0.029)].

People misdiagnosed as having T2DM tend to have a lower HbA1c at diagnosis which falls further [HbA1c falls from 5.7 to 5.3 (p < 0.001)]; and their BMI remains static, or may fall [29.2 kg/m^2^ to 28.4 kg/m^2^ (n.s)] whereas those who are correctly diagnosed as having T2DM have a higher HbA1c which falls [HbA1c falls from 7.7 to 7.2 (p = 0.004)]; but their BMI increases [28.9–29.8 (p = 0.002)].

Those who were miscoded with non-specific codes so were not on the P4P disease had poorer glycaemic control. The mean HbA1c was significantly lower in patients on the disease register (HbA1c = 7.0, SEM = 0.11 vs. HbA1c 8.1 SEM = 0.42 for the P4P vs. Non-P4P disease register group, p = 0.006).

#### Technical interface

**Figure 3:**
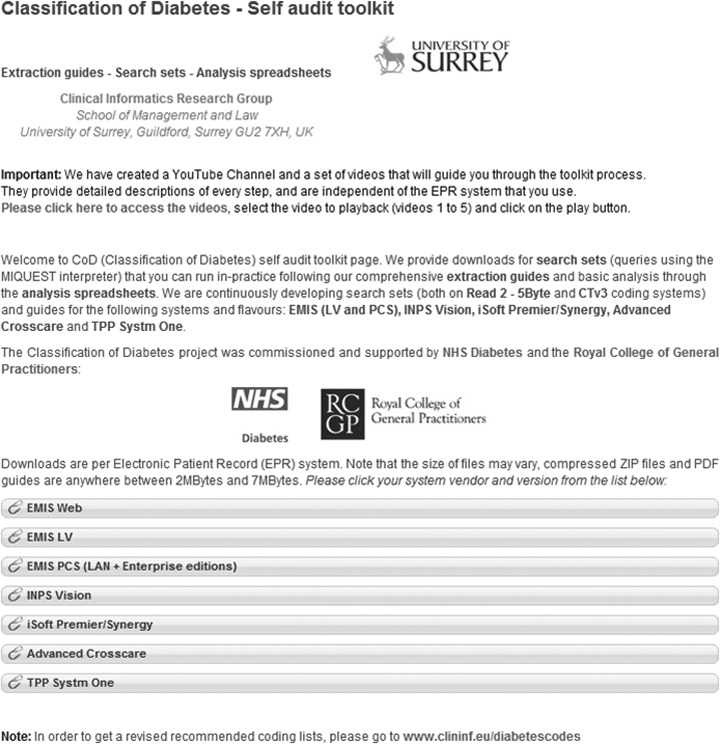
The download site for each brand of computerised medical records system (http://www.clininf.eu/cod)

The simplicity of the technical procedures involved in the audit was clear and played a central role in its success. The process of downloading and saving the search files from the internet was accessible and straightforward (Figure 3). Access to patient medical records was simple as laboratory data and prescribed medication were complete for all patients. This helped greatly when completing the audit worksheet. Extracting patient details from the data analysis spread sheet (output file of the searches) was uncomplicated and all the output files contained data that could be directly imported into the audit worksheet without accessing the medical records.

Despite the technical procedures being straightforward, there were a number of challenges which may limit its success. Running the search files was unproblematic in all but one practice. We had an issue with the search file omitting the ‘not in’ clause, which meant that a number of people who had no recorded diabetes were included in the output file. A representative of the electronic database software rectified the problem.

A number of general practitioners recorded approximate dates (to the nearest year) for diagnosis. This created challenges when establishing whether a patient had been on insulin from diagnosis or not, causing potential classification issues. Family history was also not always specified in medical records, which may affect statistical analysis on completion of the audit. The inclusion of the ‘age-bands’ heading in the results output file was also observed to be lacking, which was amended by a manual search and input of the data into the audit worksheet. Inconsistencies in the search output field for ‘reference number’ also had to be corrected in this manner.

Practice age also complicated the audit process. One of the practices started using electronic patient records a few years after set up. This meant that there was little historic computerised data, leading to searching paper medical records manually to obtain patient information. A final challenge was that of finding the first laboratory reading or the reading at diagnosis. For some patients the first reading was recorded well before diabetes was diagnosed, to overcome this, we used the reading closest to the diagnosis date.

Our experience of carrying out the audit was straightforward when the medical records were complete and the searches were functioning fully. However, an initial overview of what the audit was trying to achieve would be valuable for those who have little background knowledge of what the audit is trying to achieve. The practitioners involved in the audit found the audit easy to run given a manual. They found the process useful when monitoring the quality of care in diabetes and will incorporate it into their future practice. However, practitioners pointed out that their electronic patient data system vendor also offered tools within their computer system addressed some of the misclassification issues; and that these in-system tools were much easier to use.

The self-audit toolkits were available from three different web-sits, NHS Diabetes, Royal College of General Practitioners (RCGP), and University of Surrey Clinical Informatics group. All of these used different log-files to measure usage. The three common statistics we could obtain were: visits to the download page, unique visitors to the download pages and time taken on the download page (Table 2). Visitors would have to have navigated through at least three levels of the websites to get this far. In the six weeks after launch we had: 4235 visits; of which 3598 were unique visitors and the mean time they visited was for 2 min 55 s (a time period compatible with downloading the self-audit toolkit). However, we don’t know if they completed the download or conducted the audit.

**Table 2:** Website usage – unique visitors to the download pages

Period 01/03–15/04	Page views	Unique users page views	Average time on page
NHS Diabetes Site	2652	2296	3 min 22 sec
Clininf Site	1286	882	2 min 42 sec
RCGP Site	445	420	2 min 40 sec
TOTAL	4383	3598	2 min 55 sec

We received 10 complaints about problems with the process. All but one was resolved in 1–3 days by email (Table 3). One was because of an error with the practice computerised medical record system and solved by their software support updating the data extract system. One error was because of a person being unable to access the download screen on the Clinical Informatics website. This was caused by their work computer having an old type of web-browser which could not open the query download ‘roll-over’ menu (in more modern web browsers when the mouse rolls over the brand of computerised medical records the sub menu opens automatically). The user did not have the access rights needed to update his web-browser. We therefore created a new link to a page for people with out-of-date web-browsers (http://www.clininf.eu/cod/links). All the human error problems were solved except one. We responded to one about the layout of the audit-sheet by changing it from a column per patient to one-line per patient. We could not resolve one problem; this was where the user did not have sufficient familiarity with either spread sheets or the computerised medical record system to complete the task.

**Table 3:** Errors reported with the self-audit tools, causes of the errors and time taken to resolve

Error taxonomy	Description	Brief descriptor	Related emails	Time to resolve (days)
A	Data extraction queries and process			
B	Extraction system (translation layer/proxy)	Error in EMIS (system vendor) clinical system – user had to contact vendor	1	1
C	Top level system and database (original schema)			
D	Underlying software, networking and OS (system and communications)	Error on download website, incompatible Browser version. Users could not download the files	2	1
E	Hardware layer and infrastructure			
F	Human errors	User’s misunderstanding for the features of the toolkit (requesting process that is not provided, clarifications on manuals, explanation for location of the files, lack of proper IT skills from the user’s side	8	1(min)–3(max)
Total			12	

## Discussion

### Principal findings

The diabetes audit tools were usable. The primary problem that the audit identified was inconsistency in the quality of electronic patient record keeping. The audit highlighted to practices the need to constantly review electronically held data to improve the data quality in their records. Tools embedded within the practice EPR systems currently do not identify all the cases identified by the audit tools, however they are more familiar to practitioners and perceived as much easier to use.

The searches have utility in that they flag cases that could be better managed. People on the disease register achieve better biomedical outcomes than those not included in the register.

The results also identified typical errors: People with T2DM misclassified as T1DM are on average 15 years older and show more substantial reduction in HbA1c since diagnosis than those with T1DM. Those who are misdiagnosed as having T2DM have much lower glycated haemoglobin than those with true T2DM, mimicking excellent diabetic control.

Large numbers of people accessed the self-audit tools, were on the downloads page for periods of time compatible with downloading the toolkit, and reported very few errors.

### Implications of findings

On-line self-audit tools are usable in practice and high rates of website usage suggest that they have a good level of uptake. Direct observation of their use enables further improvement.

They have utility because they identify people who are not listed on disease registers, and those with incomplete or inaccurate data who may be receiving a lower quality of care. Practitioners should critically appraise the classification and diagnosis of diabetes; older people where control of diabetes appears to be excellent, practitioners should be aware that this may actually represent an incorrect diagnosis. Leaving people off the disease register matters as this is associated with a lower standard of care; and leaves practitioners open to criticism that they might not be including hard-to-manage cases in disease registers.

### Comparison with the literature

Other downloadable query sets exist for other medical conditions, such as Improve Access to Psychological Therapies (IAPT) (9), which have been used successfully to improve quality of care through appraisal and auditing of held electronic patient records.

### Limitations of the method

Routine data is not always complete; it is possible that more information existed for some of these patients, either in clinic letters or in free-text in these patients’ records (24). Clinical judgment is required when analysing the output data of the searches. It was also not possible to estimate the completeness of the intervention; whilst clinically important cases were detected it was not possible to know if this audit completely rectified all the possible problems with the misdiagnosis, misclassification and misdiagnosis of diabetes.

### Call for further development

The audit toolkit can be further improved with the use of the feedback system on the website (12). Vendors of electronic patient database systems should be encouraged to incorporate partially automated versions of these searches into their clinical computer system; (25) and to make it as user-friendly as possible for this type of audit to be conducted on a regular basis in practice.

## Conclusions

The downloadable self-audit toolkit is a simple and successful way of highlighting patients that may have coding, classification or diagnosis issues with diabetes. The toolkit can be run with little previous experience with the aid of the manual; but modifications and additions as a result of this evaluation should make this more user friendly. On-line downloadable toolkits appear to be a potentially effective way of rapidly stimulating quality improvement in diabetes, though they may be superseded by more effective tools built into practitioners electronic database systems. Practitioners should critically appraise how cases of diabetes are classified and where control appears excellent check the diagnosis.
